# Optimizing Decision-Making Processes in Times of COVID-19: Using Reflexivity to Counteract Information-Processing Failures

**DOI:** 10.3389/fpsyg.2021.650525

**Published:** 2021-06-22

**Authors:** Michaéla C. Schippers, Diana C. Rus

**Affiliations:** ^1^Department of Technology and Operations Management, Rotterdam School of Management, Erasmus University, Rotterdam, Netherlands; ^2^Faculty of Behavioural and Social Sciences, Organizational Psychology, University of Groningen, Groningen, Netherlands

**Keywords:** COVID-19, crisis, reflexivity, information-processing failures, groupthink

## Abstract

The effectiveness of policymakers’ decision-making in times of crisis depends largely on their ability to integrate and make sense of information. The COVID-19 crisis confronts governments with the difficult task of making decisions in the interest of public health and safety. Essentially, policymakers have to react to a threat, of which the extent is unknown, and they are making decisions under time constraints in the midst of immense uncertainty. The stakes are high, the issues involved are complex and require the careful balancing of several interests, including (mental) health, the economy, and human rights. These circumstances render policymakers’ decision-making processes vulnerable to errors and biases in the processing of information, thereby increasing the chances of faulty decision-making processes with poor outcomes. Prior research has identified three main information-processing failures that can distort group decision-making processes and can lead to negative outcomes: (1) failure to search for and share information, (2) failure to elaborate on and analyze information that is not in line with earlier information and (3) failure to revise and update conclusions and policies in the light of new information. To date, it has not yet been explored how errors and biases underlying these information-processing failures impact decision-making processes in times of crisis. In this narrative review, we outline how groupthink, a narrow focus on the problem of containing the virus, and escalation of commitment may pose real risks to decision-making processes in handling the COVID-19 crisis and may result in widespread societal damages. Hence, it is vital that policymakers take steps to maximize the quality of the decision-making process and increase the chances of positive outcomes as the crisis goes forward. We propose group reflexivity—a deliberate process of discussing team goals, processes, or outcomes—as an antidote to these biases and errors in decision-making. Specifically, we recommend several evidence-based reflexivity tools that could easily be implemented to counter these information-processing errors and improve decision-making processes in uncertain times.

*“Be open to adjustments. There’s nothing about this current moment in history that allows for stubbornness.”*

∼Unknown

## Introduction

The COVID-19 crisis has left few, if any, countries untouched and world governments have been faced with the difficult task of making decisions in the interest of public safety and health under conditions of tremendous uncertainty and time pressure. Faced with constantly changing and conflicting information, high stakes, time pressure, and a need to balance multiple concerns and interests (e.g., physical and mental health, the economy, and personal rights), governments have found themselves having to make decisions on complex issues under suboptimal conditions (Rastegary and Landy, 1993; Otte et al., 2017, 2018; cf. Schippers et al., 2007, 2015, 2017, 2018). Prior research suggests that decision-making effectiveness in highly complex and uncertain situations, such as the current crisis, largely depends on a groups’ ability to successfully acquire, integrate and make sense of information (Hammond, 1996; Schippers et al., 2014). In other words, it depends on the quality of the decision-making process which is an important prerequisite that (does not guarantee but) increases the likelihood of positive outcomes (Nutt, 1999; Bloodgood, 2011; Wolak, 2013). Importantly, while it may not be possible to determine which decisions are best, it is possible to improve the processes being used to come to those decisions, and thus increase the chances of positive outcomes (Hart, 1991).

Prior research also suggests that distortions and failures in the decision-making process are quite common (Schippers et al., 2014), especially in large decision-making groups operating under suboptimal conditions. In fact, research in large companies has found that nearly 50% of decisions fail, and one of the reasons for this is a flawed decision-making process (Nutt, 1999). Whereas a variety of different factors may influence government level decision-making processes in times of crisis (Beal, 2020; Mercer, 2020), previous research has identified a number of different biases and errors that may lead to information-processing failures. Information-processing failures consist of *“a distortion in the exchange of, communication about, or elaboration on information due to either an omission error in information sampling or biased elaboration of the information”* (Schippers et al., 2014, p. 733). For instance, in high stress situations, decision-makers have been found to rely on habit and use decision-making strategies they are most familiar with (Soares et al., 2012), a problem compounded by high time pressure (Ordóñez and Benson, 1997). In addition, framing effects and escalation of commitment may also bias the way in which information is processed (cf. Schippers et al., 2014). While these errors may readily occur at the individual level, they are often magnified in larger decision-making groups, due to additional team level biases and errors (Hinsz et al., 1997), such as, for instance, groupthink, where decisions are made based on a biased sampling of information and the focus is on agreement at all costs (Janis and Mann, 1977; Janis, 1982). Importantly, these information-processing failures have been shown to negatively impact the quality of the decision-making process (Hammond, 1996; Halpern et al., 2020).

Clearly, while the COVID-19 crisis is ongoing, it is difficult to assess the long-term effectiveness of policymakers’ decisions, not only because we currently lack the information but also because governments will have to trade off different short- and long-term concerns and interests. Yet, what is clear is that the circumstances surrounding the COVID-19 crisis are likely to make the decision-making processes more vulnerable to information-processing failures due to the high stakes, time pressure, complexity, and uncertainty involved (e.g., Joffe, 2021), thereby increasing the chances of suboptimal outcomes. Indeed, emerging evidence indicates that, physical and mental health, social cohesion, educational outcomes, economic development and human rights have all been negatively affected during this crisis (cf. Codagnone et al., 2020; Kissler et al., 2020a; for a review see Kissler et al., 2020b). Therefore, it is imperative to gain a better understanding of the potential biases and errors that might lead to information-processing failures and identify ways in which they can be mitigated. Hence, *our first aim* is to build upon and extend previous work on group decision-making processes (cf. Schippers et al., 2014) and identify what biases and errors are most likely to lead to information-processing failures in the current COVID-19 crisis. We use a theoretical framework derived from previous research on groups making complex decisions (cf. Schippers et al., 2014) and extend it to decision-making under uncertainty. Given that information about ongoing government decision-making processes is not readily available, our analysis will rely on some of the published evidence on policies implemented by governments to mitigate the COVID-19 crisis and the effects thereof. Note that we do not claim to be exhaustive in this narrative review. *Our second aim*, is to show how team reflexivity —a deliberate process of discussing team goals, processes, or outcomes—can function as an antidote to biases and errors in group decision-making. From prior research, we know that information-processing failures can be avoided and overcome, and researchers have previously suggested that an effective method for doing so is by fostering a reflexive decision-making process in groups (Schippers et al., 2014). Specifically, we will propose several simple tools that decision-making groups, such as policymakers, could use to help counteract information-processing errors and increase the chances of effective decision-making as the crisis unfolds.

We deem the contributions of this narrative review to be twofold. First, we contribute to our understanding of the biases and errors that may hamper decision-making quality and outcomes due to information-processing failures in handling the COVID-19 crisis. While not all instances of information-processing failures result in major consequences, during the current crisis, these remain a serious and potentially deadly pitfall (Schippers, 2020). Second, given that good decision-making processes enhance the chances of high-quality decisions and decision outcomes (Nutt, 1999; Bloodgood, 2011; Wolak, 2013) we show how the decision-making process can be improved via reflexivity. A reflexive decision-making process may prove particularly beneficial in the current crisis, given that it has been shown to optimize decision-making processes in groups vulnerable to information-processing failures, such as those facing complex tasks under time constraints (cf. Schippers et al., 2014, 2018). Clearly, a reflexive decision-making process, will not guarantee a positive outcome, yet, it increases the chances that the quality of the decisions made are better.

In the following sections, we will first briefly introduce our theoretical framework. Second, we will identify biases that might lead to specific information-processing errors in policymakers’ handling of the COVID-19 crisis and present practical reflexivity tools that can be used to overcome these biases. Finally, we will discuss potential policy implications, some of the limitations of our approach and make some suggestions for future research.

## Information-Processing Failures During Crisis and Reflexivity as a Potential Antidote

**FIGURE 1:**
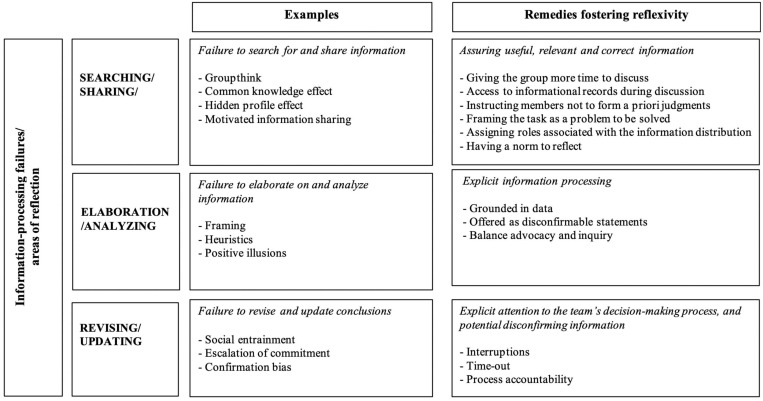
Information processing failures and remedies fostering reflexivity. Adapted from Schippers et al. (2014).

While individuals do differ in terms of decision-making competence (Bruine De Bruin et al., 2007), our focus is on the group level decision-making process. In line with prior research, we conceptualize groups as information-processing systems whose effectiveness relies on successfully sharing, analyzing, storing, and using information (cf. Hinsz et al., 1997; De Dreu et al., 2008; Schippers et al., 2014). As information-processing systems, teams are vulnerable to information-processing failures, stemming from both individual cognitive shortcomings, such as bounded rationality (e.g., Kahneman, 2003), and from breakdowns in interpersonal communication such as misunderstandings or withholding of information (cf. Hinsz et al., 1997; Schippers et al., 2014). Notably, individual-level cognitive shortcomings are often magnified in larger decision-making groups, due to further information distortion created by poor communication (Hinsz et al., 1997). In this respect, prior research suggests that groups making complex decisions are vulnerable to three specific information-processing failures: (1) a failure to search for and share relevant information; (2) if information is shared, a failure to elaborate on and analyze information; and (3) a failure to revise and update conclusions in the light of new information (cf. Schippers et al., 2014, 2018; see Figure 1 for an overview of the biases and errors which fall into these categories). Importantly, these information-processing failures have been shown to hamper groups’ ability to successfully acquire, integrate and make sense of information and are likely to increase the chances of a flawed decision-making process (Hammond, 1996; Schippers et al., 2014).

Prior research also suggests that information-processing failures can be avoided and overcome via reflexivity (cf., Schippers et al., 2014, 2018). Reflexivity is most often defined as: *“the extent to which group members overtly reflect upon, and communicate about the group’s objectives, strategies (e.g., decision-making) and processes (e.g., communication), and adapt them to current or anticipated circumstances”* (West, 2000, p. 296). Specifically, it has been proposed that team reflexivity: (1) may mitigate the failure to search for and share information by increasing the likelihood that groups will identify and use relevant and correct information (Brodbeck et al., 2007); (2) may mitigate the failure to elaborate on and draw implications from available information through explicit information-processing (cf. Lubatkin et al., 2006); and (3) may mitigate the failure to revise and update conclusions by encouraging or facilitating explicit attention to the team’s decision-making process (cf. Schippers et al., 2014; see Figure 1 for a list of potential reflexivity tools that can be used to help counteract these three information-processing failures). Crucially, reflexivity has been shown to help improve team performance (Schippers et al., 2013; Gabelica et al., 2014; Konradt et al., 2016; Lyubovnikova et al., 2017; Otte et al., 2017; Yang et al., 2020) and several review articles have examined when and why reflexivity is effective (e.g., Widmer et al., 2009; Schippers et al., 2014, 2018; Konradt et al., 2016; Otte et al., 2018).

In the following sections, we will use Figure 1 as a framework to (1) describe some examples of different biases and errors that may lead to information-processing failures in policymakers’ handling of the COVID-19 crisis, and (2) highlight specific reflexive decision-making strategies that could be used to optimize the decision-making process and minimize the occurrence of information-processing errors.

### Failure to Search for and Share Information and How Reflexivity Could Help

The first kind of information-processing error which could affect decision-making during this crisis involves a failure to search for and share all relevant information. Searching for and sharing all relevant information is especially important in situations where complex decisions need to be made based on input from multiple sources (Schippers et al., 2014), such as the handling of the COVID-19 crisis. Indeed, in the current situation, policy decisions are being made with input from multiple sources and fields (e.g., epidemiology, economics, and behavioral sciences) in order to try and maximize the information considered (Holmes et al., 2020; Romei et al., 2020), and thereby, reach the best possible conclusions. A failure to search for and share information can stem from a variety of reasons, such as a common knowledge effect, motivated information sharing or groupthink (cf. Schippers et al., 2014). In the following, we will focus specifically on groupthink, a phenomenon that has been identified as being most likely to occur during group decision-making under stress (Sterman, 2006), such as the Bay of Pigs invasion of Cuba (Janis and Mann, 1977; Janis, 1982), or the space shuttle Challenger accident (Esser and Lindoerfer, 1989). We will also propose some ways in which a reflexive decision-making process may help in mitigating some of the information-processing failures potentially stemming from groupthink.

Groupthink is a phenomenon that occurs when a group of well-intentioned people makes sub-optimal decisions, usually spurred by the urge to conform or the belief that dissent is impossible (cf. Janis, 1982). Oftentimes, these groups develop an overly narrow framing of the problem at hand, leading to tunnel vision in the search for possible solutions. Moreover, information that is not in line with or contradicting the majority view is ignored or even suppressed and there is strong pressure among group members to reach an agreement (Janis, 1991). For instance, prior research has shown that decision-making teams tend to primarily focus on discussing commonly shared information, while simultaneously minimizing discussion of unique opinions or information (Larson et al., 1996). Furthermore, group members often avoid or hesitate to share information that could cause disagreement and disturb the harmony within the group (Janis, 1991). According to researchers, groupthink often occurs when wishful thinking and reality denial start at higher levels of the organization and trickle down to become an integrated part of the decision-making process at all levels (Bénabou, 2013). Furthermore, organizational structural and procedural faults have been regularly related to groupthink (Tetlock et al., 1992).

At the beginning of the COVID-19 crisis, governments were faced with an unprecedented threat that required quick action. Early estimates stated that seven billion infections and forty million deaths could arise (Joffe, 2021) with estimates of case fatality rates ranging from 0.17% to as high a 20% (the latter was claimed in an article of Baud et al., 2020; for a review see Caduff, 2020). Moreover, early models predicted that the spread would be exponential (Banerjee et al., 2021; Ferguson et al., 2020). Based on these early estimates, many governments decided to take decisive action and enforce a combination of strict lockdowns, curfews, and the closing of “non-essential businesses” (cf. Hsiang et al., 2020; Choutagunta et al., 2021) aimed at slowing down the spread of the virus and preventing a collapse of critical care capacity. Some evidence seems to suggest that these radical policy packages deployed to reduce the rate of transmission have significantly slowed the exponential spread in certain countries such as China, Italy, France, and the United States (Hsiang et al., 2020; but also see Bjørnskov, 2021). Yet, measures exclusively focused on slowing the spread of the virus have also been linked with current and future economic decline (e.g., McKee and Stuckler, 2020) and decreased mental well-being of the general population, frontline health-care and essential workers (e.g., O’Connor et al., 2020; Robinson and Daly, 2021; Buckner et al., 2021; Toh et al., 2021; Vanhaecht et al., 2021). At the same time, the COVID-19 crisis negatively affected non-Covid related public health such as the postponement or cancelation of medical treatments (Heath, 2020; Schippers, 2020). Also, the policies have exacerbated existing human rights violations in many countries, and enabled others (Fisman et al., 2020; Saunders, 2020). Thus, it appears that an initial focus on slowing the spread of the virus may have led to a narrow problem framing, which may have resulted in either discounting information about, or minimizing the possible extent of negative consequences in other domains, such as the economy, well-being, non-Covid related public health, or human rights. Some researchers have, for instance, suggested that little attention has been paid to the potential side effects of the preventative measures taken, and questioned the extent to which some countries’ policies are evidence-based and proportional (Ioannidis, 2020; Ioannidis et al., 2020; Schippers, 2020; Joffe, 2021). A narrative review of Joffe (2021; p. 1) concluded that the cost-benefit analysis of the COVID-19 response was very negative and that “lockdowns are far more harmful to public health than COVID-19 can be.”

Relatedly, given that most governmental policies have been grounded in the precautionary principle (Sunstein, 2019) of avoiding deaths and minimizing the spread of the virus, the communication of these policies has tended to rely on war analogies and fear-based references to the magnitude of the threat to justify a “one size fits all” approach (Caduff, 2020). In the process, it appears that dissenting voices may have been drowned out in various countries ranging from Western liberal democracies to more autocratic states (cf. Abazi, 2020; Niemiec, 2020; Sherman, 2020; Timotijevic, 2020). For instance, the mainstream public discourse has largely ignored early voices suggesting that lockdowns might significantly disrupt supply chains, lead to massive unemployment, and to exacerbating poverty in developing countries leading to food insecurity for more than 100 million people (Inman, 2020; Zetzsche and Consiglio, 2020). Also, in some countries, those questioning the measures were silenced, marginalized or labeled as traitors in the mainstream media (Abazi, 2020; Joffe, 2021). Although very worrisome, this is in line with previous work suggesting that silencing dissenting opinions is a historically common government response to pandemic situations, aimed at steering the public narrative and bolstering support for government actions (Timotijevic, 2020). In addition, given the proliferation of fake news and misinformation, many technology platforms have been forced to rush in and remove potentially dangerous false information (Abrusci et al., 2020). Yet the censorship of social media as a remedy to the spread of medical disinformation has been called into question (cf. Niemiec, 2020) and some evidence suggests that simple nudging interventions might also work in fighting misinformation, without the need for pervasive social media censorship (cf. Pennycook et al., 2020). Whereas presenting a strong, united front in the face of possible panic is important, it is equally important to allow for dissenting and conflicting opinions to be brought forward. This is all the more important in situations such as the current crisis, where potentially relevant information is spread across multiple disciplines and the state of knowledge is constantly evolving and changing. In this respect, some authors have highlighted a lack of access and transparency regarding the data used by policymakers, poor data input and a reluctance to admit uncertainties in the data (Heneghan and Jefferson, 2020; Ioannidis et al., 2020; Jefferson and Heneghan, 2020), selective reporting of forecasts, and a lack of transparency in the modeling and assumptions used to inform public policy (Ioannidis et al., 2020). These may all have impeded building an accurate understanding of the situation based on shared facts and open public discourse among different groups of scientists and policymakers.

Importantly, ignoring or silencing dissenting and conflicting opinions is likely to induce groupthink and lead to a narrow focus in the decision-making process during crisis. This, in turn, has been shown to lead to decisions based on incomplete or one-sided information, which negatively affect the chances of achieving positive outcomes (Hart, 1991). In this case, the failure to search for and share as much relevant information as possible may also have been compounded by a general human tendency to underprepare for disasters (Meyer and Kunreuther, 2017; Murata, 2017), and the fact that warnings from the scientific community to plan for a potential deadly viral outbreak before the COVID-19 crisis were repeatedly ignored (Horton, 2020). Thus, without a clear response plan, as the crisis emerged, many governments were under pressure to rapidly make sense of incoming information, reach quick decisions, and take decisive action. This pressure may have been amplified by a fear of being blamed for doing “too little” (Bylund and Packard, 2021) and by the intense media focus on the issue. Consequently, initially exaggerated pandemic estimates, case fatality rates, projected rates of community spread, and a focus on only a few dimensions or outcomes at the expense of the larger picture (cf., Ioannidis, 2020; Ioannidis et al., 2020), may have led to some wrong assumptions underlying initial pandemic-response policies. Furthermore, these assumptions may not have subsequently been questioned or updated based on newly emerging information.

In sum, while the COVID-19 situation is still unfolding, it is difficult to ascertain whether groupthink is indeed featuring in individual government’s decision-making processes, yet, based on our analysis, it is possible that at least some of its characteristics might occur (see also Timotijevic, 2020; see Joffe, 2021 for examples of groupthink). Clearly, at this point in time, neither the evolution of the disease itself nor the long-term economic, societal, mental health or human rights impact of the crisis can be known. Although some researchers have attempted to predict how events will unfold (McKibbin and Fernando, 2020), it is still too early to understand what the long-term effects will be. That being said, there seems to be some evidence suggesting that a long-term public policy exclusively focused on slowing the spread of the virus does have negative side-effects in society at large, some of which may have been avoidable via a more holistic approach integrating multiple perspectives and points of view. A holistic approach integrating information from multiple sources, perspectives and points of view has been shown to be critical in ensuring a better quality of the decision-making process (cf., Schippers et al., 2014).

In this respect, we propose reflexivity as a method of counteracting reliance on incomplete information, as it explicitly encourages the pooling and consideration of information scattered across multiple group members (Schulz-Hardt et al., 2006). Reflexivity encourages making the decision-making process an explicit balance of advocacy and inquiry, with a focus on widening the array of opinions considered, rather than on decision-making harmony within the group (for an overview of some practical tips for fostering reflexivity, see Figure 1). For instance, one practical tool that may offer a simple solution to counter groupthink is the use of a simple checklist (see Table 1). This checklist is based on the early work on groupthink by Janis (1991) and forms a useful basis as a quick screen for symptoms of groupthink to be aware of, check for, and avoid. Furthermore, previous research suggests that actively encouraging the discussion of unique, or dissenting opinions is also important, as it allows for a broader framing of the problem at hand and protects against the pitfall of groupthink (cf. Emmerling and Rooders, 2020). In order to facilitate the open sharing of information, previous research suggests that creating psychological safety within the group (cf. Edmondson, 1999) and appointing a strategic dissenter are critical (Emmerling and Rooders, 2020). Moreover, transformational leadership (Schippers et al., 2008) and avoiding an overreliance on experts (Gino and Staats, 2015) have also been shown to facilitate reflexive decision-making processes likely to incorporate a broader array of information, interests and perspectives.

**TABLE 1:** Overview of checklist items to ensure minimization of groupthink.

◻	Allowing team members the chance to critically assess the actions of the group and promotes criticism of his judgments.
◻	The leader/manager is impartial and does not state their personal opinions, especially at the beginning of the discussion.
◻	When a complex problem must be addressed, the team works it out in parallel groups, and then returns to discuss it as a whole afterward.
◻	When evaluating the feasibility and effectiveness of certain decisions, the group occasionally splits into two or more subgroups for discussions.
◻	Each group member regularly discusses the direction of the group with third parties from outside the team, and seeks feedback on the group process.
◻	Outside experts are invited to contribute to the discussion.
◻	A group member is assigned to the role of “devil’s advocate “during meetings, and their role is to highlight the disadvantages of any discussed actions, in order to promote the discussion about consequences.
◻	Organize a second chance assessment, in which after reaching a provision consensus, group members will still get the chance to consider a second opinion, with a chance for reconsideration.

*Adapted from Janis and Mann (1977).*

### Failure to Elaborate on and Analyze Information and How Reflexivity Could Help

Even if (reliable and high-quality) information has been gathered, information-processing failures can occur during the process of analyzing and elaborating on that information. Prior research suggests that information elaboration is especially critical in highly turbulent times (Resick et al., 2014) and when groups are faced with a complex task (cf. Vashdi et al., 2013; Schippers et al., 2014), such as the current COVID-19 crisis. Failures to elaborate on and analyze the implications of available information can stem from a variety of reasons, the most important ones being framing effects (i.e., the tendency to make different decisions based on how the problem is presented; Tversky and Kahneman, 1981), reliance on heuristics (i.e., simple rules of thumb guiding decisions; Kahneman, 2003), and positive illusions, such as for instance, illusions of control (cf. Schippers et al., 2014; Figure 1). In the following, we will focus specifically on how framing effects may lead to errors in analyzing and elaborating on the available information in handling the COVID-19 crisis, and we will propose some ways in which a reflexive decision-making process may help in mitigating these errors.

Framing effects occur when presenting information in different ways changes, and even reverses, how people make decisions about equivalent choice problems (e.g., Kahneman, 2003). Prior research suggests that framing influences both problem definition and causal analysis (cf. Entman, 2007). As such, framing effects have been shown to be critical to our understanding of how people make decisions, especially decisions involving risk (for recent meta-analyses see Kühberger, 1998; Steiger and Kühberger, 2018). In addition, recent research suggests that time pressure amplifies framing effects (Diederich et al., 2018), especially in group-decision-making settings, due to group polarization (i.e., groups show a pronounced tendency to shift to more extreme positions than those originally held by any of the individual members; Cheng and Chiou, 2008). The first demonstration of the framing effect stems from an experiment by Tversky and Kahneman (1981), who used an experimental paradigm, the ‘Asian Disease Problem,’ to test how the framing of a problem in terms of potential gains and losses affected decisions about possible solutions. In this experiment, participants are given a scenario in which they are warned about the outbreak of a dangerous disease, expected to kill 600 people. Then they are presented with a choice between two equivalent solutions (one involving a certain outcome and the other involving a risky outcome), which are framed either as a gain (lives saved) or as a loss (lives lost). When participants were presented with solutions framed as a gain (number of lives saved), they tended to choose the solution with a certain outcome. However, when they were presented with solutions framed as a loss (number of lives lost), they tended to choose the solution with a risky outcome. This study which has been replicated in various contexts (cf. Steiger and Kühberger, 2018 for a recent meta-analysis), including during the COVID-19 pandemic (Hameleers, 2020), suggests that framing a decision in terms of numbers of lives lost (vs. saved) tends to lead to decisions involving higher risks.

These findings might be highly relevant during the COVID-19 crisis, which has been characterized by extensive social and popular media coverage, overwhelmingly focusing on the daily infection rates, hospital occupancy rates, and virus-related death toll (cf. Ogbodo et al., 2020; Schippers, 2020). This incessant media focus on tracking daily infections and lives lost and framing the discourse as a choice between public health and the economy (cf. Codagnone et al., 2020; Huseynov et al., 2020), has also contributed to shaping public opinion and the spreading of fear (Ogbodo et al., 2020). In addition, it may even have influenced various policy choices, which would be in line with past research showing that media coverage of health emergencies (e.g., epidemics and pandemics) has been crucial in the framing of public policy debates and policy responses (Karnes, 2008; Dry and Leach, 2010; Pieri, 2019). Thus, given the overwhelming public focus on the daily reports of new infections and deaths, policymakers might have felt pressured to make quick decisions based on these rapid number fluctuations. Relatedly, the problem has tended to be framed narrowly as one of avoiding deaths caused by the new coronavirus, as opposed to being framed more broadly as one of public health, or even more broadly as one of societal well-being — with all that it entails, including a healthy economy, public physical and mental health, social justice, etc. This narrow problem framing, in turn, may have influenced information elaboration and analysis of the situation and, paradoxically, may have led to riskier policy decisions (cf. Ioannidis, 2020) than a broader problem framing would have.

For instance, a focus on preventing COVID-19 related deaths has led to a number of policies centered around containment, which have included the controversial closing of borders and shutting down of entire societies for weeks or even months (for some criticisms regarding the evidence-base of such decisions see Ioannidis, 2020; Ioannidis et al., 2020). Whereas these policies may have indeed reduced individuals’ risk of infection, they also exposed them to other risks, such as losing their sources of livelihood (e.g., Codagnone et al., 2020), depression, burnout, and anxiety (e.g., Amerio et al., 2020; Fiorillo et al., 2020; O’Connor et al., 2020; Robinson and Daly, 2021; Buckner et al., 2021). It also appears that vulnerable populations such as those already suffering from mental health issues or addictions, and women and children living in abusive households may have been particularly negatively affected (e.g., Serafini et al., 2016; Buttell and Ferreira, 2020; Clarke et al., 2020; Graham-Harrison et al., 2020; Pfefferbaum and North, 2020; Reger et al., 2020; Schippers, 2020; Zetzsche and Consiglio, 2020; Acenowr and Coles, 2021; Rumas et al., 2021; Sakamoto et al., 2021). It is undeniably crucial that policymakers should focus on protecting public health by preventing coronavirus-induced deaths. Yet public health can also be threatened by reduced mental well-being, the discontinuation of regular care and food insecurity. Moreover, societal well-being depends on functioning economies, the rule of law and social justice (cf. Drucker, 2003). Therefore, the main criticisms that have been brought forward have centered around the use of interventions without full consideration of the evidence pointing to their impact on society at large (Haushofer and Metcalf, 2020). A broader problem framing in terms of societal well-being might have avoided some of these negative effects, since it would have led to the consideration and balancing of a larger array of factors and interests in the decision-making process. For instance, by simultaneously taking into account effects on public, economic, and mental health, as well as on those most vulnerable in society, more evidence-based policies could have been implemented that would also have minimized risks in these domains.

The framing of the speed of spread of the virus in terms of daily exponential growth rates in the popular media is also likely to have shaped public opinion and policymakers’ decision-making processes. For instance, a pervasive bias that is highly vulnerable to framing effects is exponential growth prediction bias, the phenomenon whereby people underestimate exponential growth when presented with numerical information (Wagenaar and Sagaria, 1975; Wagenaar and Timmers, 1979). In the context of COVID-19, this bias has been shown to lead to a systematic tendency to underestimate the number of COVID-19 cases or fatality rates in the future based on current numbers (Wagenaar and Sagaria, 1975; Banerjee et al., 2021). This bias, may also have contributed to more risky decision-making, by potentially leading to unwarranted lax policy-measures (e.g., when current infection rates were low but likely to grow exponentially) or to the late introduction of stricter policy-measures (e.g., when current infection rates were already too high). In this respect, previous research has shown that a different framing and communication of exponential growth functions in terms of doubling times rather than in terms of case growth and daily exponential growth rates tends to decrease exponential growth prediction bias (cf. Schonger and Sele, 2020) and can improve the quality of the decision-making process by leading to a more accurate analysis of the data at hand.

In sum, it appears that various framing effects in the public discourse may have negatively impacted policymakers’ information elaboration and analysis of the potential implications of policies. Clearly other information-processing failures in the elaboration of information may stem from a variety of other individual-level cognitive biases, such as the availability bias or the salience bias (Kahneman, 2003; for a discussion of other specific decision-making biases that may have played a role in the handling of the COVID-19 crisis see Halpern et al., 2020) and we do not claim to be exhaustive here. Our analysis does, however, indicate that, given the complexity and uncertainty of the situation, there is a need to focus on a decision-making process grounded in data and, whenever possible, prior evidence. Of course, as the situation continues to unfold information and data at any point in time is limited and constantly being updated. Yet, a decision-making process that frames the problem to be solved more broadly and explicitly considers and weights possible consequences for a variety of societal stakeholders is critical in avoiding unnecessary risks to the health, well-being, and livelihoods of individuals.

In this respect, reflexive decision-making might help in mitigating the failure to elaborate on and analyze the implications of one’s decision-making (cf. Schippers et al., 2014). A reflexive decision-making process can help in terms of facilitating data-driven decisions and highlighting the need to create disconfirmable statements (i.e., phrased in such a way that they are falsifiable). This would facilitate deliberate reflection by allowing for discussions that balance advocacy and inquiry, a careful weighting of the information available, and the consideration of different stakeholders’ perspectives (see Figure 1), thereby aiding a group in creating a realistic picture of the situation. For instance, one possible way to facilitate deliberation and a decision-making process grounded in data would be to apply strategies aimed at minimizing framing effects. Some evidence-based strategies that could easily be applied by policymakers are, for example, multitracking and considering multiple frames simultaneously (e.g., saving lives *and* saving the economy vs. saving lives *or* saving the economy); broadening the frame (e.g., focusing on societal well-being rather than on solely avoiding COVID-19 related deaths); increasing the number of options or solutions considered simultaneously; shifting one’s reference point (e.g., shifting from a prevention focus which aims at avoiding negative outcomes to a promotion focus which aims at approaching positive outcomes); and considering the opportunity costs of any particular decision (cf. Ariely, 2008; Heath and Heath, 2013). Another potentially useful technique that has been shown to facilitate deliberation, information sharing, and a weighting of relevant information in the decision-making process is brainwriting (e.g., Paulus and Yang, 2000; Heslin, 2009). In contrast to engaging in a group-brainstorming session (which typically happens in decision-making groups and has repeatedly been shown to lead to lower quality ideas; cf. Paulus and Brown, 2007), brainwriting implies that the different group members individually write down and share their ideas by passing notes to each other, prior to engaging in a group discussion. This process has been shown to be more effective than a traditional group-brainstorming technique in terms of yielding higher quality ideas, given that it allows for explicit attention to the exchanged ideas as well as providing the opportunity for group members to reflect on the exchanged ideas after they have been generated (cf. Paulus and Yang, 2000).

### Failure to Revise and Update Conclusions and How Reflexivity Could Help

Even if decision-making groups succeed in successfully elaborating on and analyzing the information available to them, effective information-processing may be compromised by a failure to revise and update conclusions. Prior research suggests that this is a particular challenge for groups making decisions in high-stakes, continuously evolving complex situations (cf. Schippers et al., 2014) such as the current COVID-19 crisis. Failures to revise and update conclusions can stem from a number of reasons (see Figure 1) such as social entrainment (i.e., the failure to update conclusions that are taken for granted due to entrenched patterns; Schippers et al., 2014), escalation of commitment (i.e., persisting on a course of action, even though changing to a new course of action would be advantageous; Sleesman et al., 2018), and confirmation bias (i.e., actively seeking out evidence that confirms one’s beliefs and expectations, while ignoring or failing to seek out evidence that might disconfirm one’s beliefs; Nickerson, 1998). Below we will discuss how escalation of commitment and confirmation bias may lead to information-processing failures in revising and updating conclusions in handling the COVID-19 crisis and propose some ways in which reflexivity could help in mitigating some of these failures.

As the COVID-19 crisis is still evolving, it is key that decision-making groups remain flexible, and are able to evaluate and change their course of action if it turns out to be necessary (Whitworth, 2020). Indeed, prior studies have shown that in order to function effectively, it is crucial that decision-making groups are able to adapt to new information and circumstances (LePine, 2005). However, this is more problematic than it seems, partly because the difficulty of their goal is often inversely related with their likelihood of successfully adapting to changing circumstances (LePine, 2005). A common bias impeding flexibility is escalation of commitment, where people keep investing more resources in a set course of action, even in the face of clear evidence that it is not working, or that better options are available (Arkes and Blumer, 1985; Dijkstra and Hong, 2019; for a review see Sleesman et al., 2018). A recent review suggests that an explanation for this phenomenon in groups lies in the need to publicly stand by and justify prior decisions, and that this tendency is magnified in diverse groups (Sleesman et al., 2018). For instance, in the context of COVID-19, it seems that early predictions on infection fatality rates (e.g., Ferguson et al., 2020), that are now known to be far too high, have hardly led to an update in policies for most countries (but see Bylund and Packard, 2021 for an account of how Swedish policymakers revised and updated their policies). The actual inferred infection fatality rates seem to be much lower than early estimates, even for countries that had light or no lockdowns (Ioannidis et al., 2020; Jefferson and Heneghan, 2020; Bylund and Packard, 2021). As a case in point, while the early prediction for California was that at least 1.2 million people over the age of 18 would need a hospital bed, and that 50,000 additional hospital beds were needed, at the height of the infection well under five percent of hospital beds were occupied by COVID-19 patients (Ioannidis et al., 2020). In the end, very few hospitals were overwhelmed, and if they were, this was only for a short period of time. In addition, it seems that early modeling for the resurgence of the virus (second and third waves) was also inaccurate (Ioannidis et al., 2020; but see Andrew, 2020 for a critique), and it has even been argued that the repeated lockdowns were too late or too loose to be effective (Chaudhry et al., 2020). The most recent study noted that the “available evidence suggests average global IFR of ∼0.15% and ∼1.5–2.0 billion infections by February 2021 with substantial differences in IFR and in infection spread across continents, countries and locations” (Ioannidis, 2021, p. 1, IFR = Infection Fatality Rate). Despite these evolving insights suggesting for instance that early intervention might be important (Dergiades et al., 2020; Chernozhukov et al., 2021), it appears that few countries critically assessed the effectiveness and timing of specific policies and changed course of action accordingly.

This potential escalation of commitment might be due to the fact that the crisis is unfolding ‘live’ under tremendous amounts of public and media scrutiny. Thus, policymakers might feel pressured to be seen as competently and decisively handling the crisis, which might lead them to stick to and justify prior decisions (cf. Sleesman et al., 2018). For instance, prior research suggests that, in crisis situations, followers expect leaders to provide clarity of direction and make things happen (cf. Sutton, 2009; Boin et al., 2013). The media reporting of the COVID-19 crisis focusing on daily fluctuations in infection rates, hospital bed occupancy and fatality rates, magnifies fear and anxiety among the general public, and thus puts pressure on policymakers to provide clarity of direction by sticking to a chosen course of action. In addition, public framing of the situation as a “war against an invisible enemy” (Wicke and Bolognesi, 2020) and the highly moralized public discourse dividing people into “patriots” and people to blame (Caduff, 2020), may also contribute to an action-oriented focus on “defeating this enemy” and an overestimation of the extent to which the situation can be controlled. This combination of public scrutiny, perceived need to provide clarity of direction and an action-orientation, leave little room for revising and updating conclusions and changing strategy.

Relatedly, confirmation bias may also have contributed to escalation of commitment and a failure to update and revise information and conclusions during the COVID-19 crisis. A tendency to focus on information in line with one’s initial ideas at the expense of disconfirming information, could lead to overreliance on interventions that are not evidence-based (cf. Ioannidis, 2020), and to the suppression of dissenting voices (cf. Abazi, 2020). This, in turn, could lower the chances of learning new information and updating conclusions. Given the uncertain nature of the situation, it is to be expected that decisions made at any given point in time may no longer be the best decisions as the situation continues to change and evolve (Tolcott et al., 1989). For instance, the most commonly implemented policy-measures are predicated on social distancing, based on the initial assumption that the primary virus transmission vector is via large droplets. However, more recent evidence seems to suggest that airborne transmission (i.e., via smaller droplets) plays a significant, yet previously underestimated, role in the spread of the virus (cf. Buonanno et al., 2020; Bazant and Bush, 2021). These new insights render policies based primarily on social distancing measures insufficient to curb the spread of the virus and would require policy revisions. Other researchers have asked for more nuanced recommendations on the use of masks by the general public given that they have potential physical and psychological side-effects (for a meta-analysis see Kisielinski et al., 2021), while others have argued for “multi-prolonged population-level strategies” (Alwan et al., 2020). Yet other researchers have called for alternative approaches which conceptualize public health in broader terms than simple infection control (Lenzer, 2020). For example, three eminent epidemiologists and public health experts from Harvard, Oxford and Stanford published the Great Barrington Declaration, which has been signed by hundreds of thousands of concerned citizens, and tens of thousands of medical practitioners and scientists arguing for a focused protection approach to handling the crisis. This proposed approach aims to balance the need to protect high-risk individuals from COVID-19 while reducing the “collateral harms” and serious consequences ensuing from prolonged lockdowns (Lenzer, 2020).

A failure to incorporate new evidence and insights into policymakers’ decision-making process can have damaging consequences not only in terms of effectively handling the public health crisis, but also in terms of potential long-term side-effects such as weakened economies, compromised democracies, and even a legitimization of the use of force (Caduff, 2020; Schippers, 2020; Wicke and Bolognesi, 2020; Zetzsche and Consiglio, 2020). We propose that reflexivity can help mitigate the failure to revise and update conclusions by facilitating explicit attention to the decision-making process (see Figure 1). We also deem it to be crucial in promoting evidence-based solutions that incorporate newly emerging scientific insights regarding the spread of the virus, potential mitigation or treatment options, and the effects of current policies. As such, reflexive decision-making is an ongoing process: groups constantly reassess the situation, collect and weigh newly arising evidence, are willing and able to reflect on the actions they have taken, and, when necessary, are prepared to change the current direction or make adjustments to it (cf. Schippers et al., 2014). For instance, an effective intervention that can promote reflexivity and help avoid escalation of commitment, is a simple reminder to “stop and think” (cf. Okhuysen, 2001; Schippers et al., 2014). This simple instruction serves as an interruption and provides some much-needed distance from action. In addition, holding groups accountable for the decision-making process (i.e., having to account for the manner in which decisions are reached) as opposed to holding them accountable for the outcomes of decisions, has been shown to facilitate more careful information-processing (cf. Lerner and Tetlock, 1999), reduce the chances of escalation of commitment (Schippers et al., 2014), and induce more complex decision-making strategies (Tetlock and Kim, 1987). A focus on process accountability as opposed to outcome accountability might be especially relevant during the COVID-19 crisis, given that the situation is highly uncertain and requires the careful consideration of multiple perspectives as well as a continuous reassessment of potential courses of action. Finally, some effective strategies that could help beat the confirmation bias trap are: seeking out information from a broad range of sources; actively seeking out disconfirming information; entertaining or testing multiple hypotheses simultaneously; sparking constructive disagreement; assigning one team member the role of devil’s advocate; or testing assumptions in small pilots prior to full solution rollout (e.g., Ariely, 2008; Bazerman and Moore, 2008; Heath and Heath, 2013). In sum, as new information becomes available, and more widespread knowledge of the effects of the crisis become visible, it is crucial that policymakers try to avoid information-processing failures by engaging in an ongoing process of reassessing the situation, incorporating newly arising evidence, and being willing to change course of action based on the evidence.

## Discussion

The Covid-19 crisis currently sweeping the globe has brought about numerous unforeseen difficulties and problems. Policymakers are making high stakes decisions about how to respond on the basis of constantly evolving and incomplete information, under time constraints, and in the face of immense uncertainty and public pressure. These suboptimal circumstances render decision-making processes vulnerable to errors and biases in the processing of information, thereby increasing the chances of faulty decision-making processes with poor outcomes. In the current situation, errors and biases in decision-making have the potential to result in widespread societal damages (Caduff, 2020; Schippers, 2020; Joffe, 2021), and it is vital that policymakers take steps to maximize the quality of the decision-making process (Halpern et al., 2020) and increase the chances of positive outcomes as the crisis goes forward.

Prior research on the effects of information-processing failures has suggested that these can be mitigated through reflexivity, however, it has not yet been explored how reflexivity can contribute to optimizing decision-making processes during times of crisis. Thus, we applied and extended the theoretical framework of Schippers et al. (2014) on information-processing failures in groups, (1) to further our understanding of the biases and errors that may hamper decision-making quality in handling the COVID-19 crisis and (2) to outline how reflexivity can help in mitigating these potential errors. In our analysis, we classified potential errors and biases as falling into one of three categories of information-processing failures: (1) a failure to search for and share relevant information; (2) if information is shared, a failure to elaborate on and analyze information; and (3) a failure to revise and update conclusions in the light of new information (cf. Schippers et al., 2014, 2018). Specifically, we identified groupthink, framing effects, and escalation of commitment as posing the largest risks to decision-making processes in handling the COVID-19 crisis and have provided practical reflexivity tools that can be used to overcome these biases.

### Implications for Policymaking

Groupthink, a narrow focus on the problem of containing the virus, and escalation of commitment pose real risks to decision-making processes in handling the COVID-19 crisis and may result in devastating consequences for lives and livelihoods for decades to come (Caduff, 2020; Schippers, 2020; Joffe, 2021). With the crisis already in full swing, information-processing failures may have already had an impact on decisions made (Halpern et al., 2020). Therefore, it is critical that future decisions are based on sound decision-making processes. To this end, we have proposed that reflexivity, may offer the key to helping policymaking groups improve their decision-making process. Implementing a reflexive decision-making process could help policymakers going forward by minimizing the occurrence of information-processing errors and by enabling them to maximize the chances of good outcomes in the future. We have recommended several evidence-based reflexivity tools that could easily be used to counter these specific information-processing errors (see Figure 1). For instance, using a checklist to assess symptoms of groupthink; appointing a strategic dissenter; creating psychological safety for speaking up; and avoiding overreliance on experts (cf. Gino and Staats, 2015; Emmerling and Rooders, 2020), could all help avoid the pitfall of groupthink. In addition, we have proposed reflexivity tools that would facilitate a broader framing of the current problem and help groups take data-driven decisions, based on a careful weighting of information and the consideration of potential consequences across different domains for various stakeholders. For example, brainwriting; multitracking and considering multiple frames simultaneously; increasing the number of options or solutions considered simultaneously; and considering the opportunity costs of any particular decision, could all help in minimizing framing effects (cf. Heath and Heath, 2013; Schippers et al., 2014) and result in more holistic policy approaches. Finally, The simple yet effective reflexivity tools we have put forward may help focus policymakers’ explicit attention to the decision-making process and help them avoid escalation of commitment, such as a simple reminder to “stop and think” (cf. Okhuysen, 2001) and process accountability.

The current pandemic has certainly been unprecedented and disruptive on all fronts. Yet, the future is likely to harbor many more unpredictable, unprecedented, highly disruptive, global events which will require quick action based on a sound decision-making process. To increase the chances of handling such future crises successfully, it is critical that policymaking groups lay the foundations for sound decision-making processes in the future by building internal capabilities in sensing, shaping, and flexibly adapting to circumstances as they happen. In other words, it is crucial that they build overall group reflexivity and reflexive decision-making capabilities. Prior research has developed several tools and interventions to help increase overall team reflexivity, which might be relevant in this respect (cf. Schippers et al., 2007; Otte et al., 2017). For instance, institutionalizing guided reflexivity processes (i.e., debriefing or post-mortem analyses), analyzing one’s own and other groups’ failures has been shown to help groups improve decision-making processes and outcomes (cf. Ellis et al., 2014; Schippers et al., 2014). Therefore, it is imperative that policymakers critically evaluate the outcomes of their and their peers’ decisions in handling the current crisis and draw learnings for the future. Evidently, in the case of unprecedented events it is impossible to reflect on and analyze past successes and failures, yet it is possible to prepare for plausible even if seemingly unlikely future events. Hence, to build capability in managing uncertainty it is also important to institutionalize reflexive group processes aimed at foresight, by using tools such as ‘premortems’ (i.e., identifying the causes of hypothetical future failures), contingency planning (i.e., creating a playbook for emergency cases), or scenario planning (i.e., using stories about possible alternative futures to challenge and reframe assumptions about the present; cf. Scoblic, 2020). Although such preparedness seems to have been available in the form of “event 201,” an exercise organized by the Johns Hopkins Center for Health Security in partnership with the World Economic Forum and the Bill and Melinda Gates Foundation. It was a high-level pandemic exercise, modeling a fictional Corona pandemic, and was aimed at diminishing societal and economic consequences^1^. When the crisis occurred, these aims seem not to have been reached, despite the uncanny resemblance of the event and the subsequent crisis. Using a scientific approach to handling these crises, this would allow for better upfront preparedness in handling future crises and facilitate an ongoing reflexive decision-making process.

### Implications for Research

Our analysis provides an important starting point in identifying potential biases and errors that may hamper the decision-making process during the COVID-19 crisis, yet it also suffers from some important limitations that warrant further investigation. First, given that the situation is currently unfolding, there is little available evidence regarding the decision-making processes that policymakers have implemented, as the process is often not transparent. Therefore, we relied on the limited published evidence on decisions made and their outcomes. Yet, it is very difficult to infer how decisions were made on the basis of their outcomes. Therefore, as more information becomes available, future research would benefit from examining what decision-making processes were used by various policymaking groups during this crisis, which processes resulted in the best outcomes, and how these processes can be implemented for use in future crisis decision-making. Second, to date, we do not have a clear understanding of the extent to which policymakers across different countries have involved the general public in the decision-making process. Based on the currently available data it appears that open public debate was shunned in numerous countries (cf. Abazi, 2020; Sherman, 2020; Timotijevic, 2020), yet it is possible that this was not the case in others. Prior research suggests that, when it comes to complex policy decisions, people care about having voice (i.e., have the opportunity to express their opinions in the decision-making process, even if not personally involved in the process). Importantly, voice has been shown to lead to increased trust in government and policy acceptance (cf. Terwel et al., 2010). Thus, investigating the extent to which the general public was given voice in the decision-making process surrounding COVID1-19 and how this may have affected policy acceptance and compliance, could provide valuable insights for engendering public support in the handling of future crises.

Third, given the limited published record on the effects of the crisis, it is possible that information on policies and their effects in certain countries may be overrepresented and too little data may be available for other countries. However, countries varied in the types and combination of measures implemented, the timing thereof, and in public compliance rates (cf. Bylund and Packard, 2021). It is therefore possible that specific combinations of measures in policy packages, their timing, and cultural differences in terms of trust in government, interact in predicting public compliance and policy outcomes. Therefore, as more information becomes available, future research would benefit from engaging in more fine-grained analyses that take into account not only the decision-making process but also such possible interactive effects. This is critical in distilling learnings from the current crisis that could provide a solid evidence-base for handling future crises. Finally, our review is not exhaustive as our main intent was to provide a framework for identifying potential errors and biases in the decision-making processes surrounding the COVID-19 crisis. As more evidence becomes available, future research would benefit from engaging in a systematic review of policymakers’ decision-making processes and their outcomes.

### Conclusion

In the current crisis, the risk of biases and errors in policymakers’ decision-making processes has the potential to cause widespread societal damages. We identified, groupthink, a narrow focus on the problem of containing the virus, and escalation of commitment as posing real risks to decision-making processes in handling the COVID-19 crisis. Hence, it is vital that policymakers take steps to maximize the quality of the decision-making process and increase the chances of positive outcomes as the crisis goes forward. Implementing a reflexive decision-making process could help policymakers going forward by minimizing the occurrence of information-processing errors and by facilitating the emergence of more holistic approaches that balance a variety of concerns, such as public (mental) health, the economy, and human rights.

## Author Contributions

All authors provided substantial contributions to the conception or design of the work, were responsible for drafting the work or revising it critically for important intellectual content, approved the final version of this manuscript, and agreed to be accountable for all aspects of the work.

## Conflict of Interest

The authors declare that the research was conducted in the absence of any commercial or financial relationships that could be construed as a potential conflict of interest.

## Footnotes

1 https://www.centerforhealthsecurity.org/event201/
